# Measurement, Collaborative Learning and Research for Sustainable Use of Ecosystem Services: Landscape Concepts and Europe as Laboratory

**DOI:** 10.1007/s13280-012-0368-0

**Published:** 2013-03-10

**Authors:** Per Angelstam, Michael Grodzynskyi, Kjell Andersson, Robert Axelsson, Marine Elbakidze, Alexander Khoroshev, Ivan Kruhlov, Vladimir Naumov

**Affiliations:** 1 Faculty of Forest Sciences, School for Forest Management, Swedish University of Agricultural Sciences, PO Box 43, 730 91 Skinnskatteberg, Sweden; 2 Department of Physical Geography and Geoecology, Taras Shevchenko National University of Kyiv, 2 Glushkov Avenue, GSP-680, Kiev, Ukraine; 3 Department of Physical Geography and Landscape Science, Moscow Lomonosov State University, 1 Leninskiye Gory, GSP-1, 119991 Moscow, Russia; 4 Faculty of Geography, Franko University of Lviv, Doroshenka Street 41, 79017 Lviv, Ukraine

**Keywords:** Sustainability, Landscape concepts, Social–ecological system, Case study, Collaborative learning, Transdisciplinary

## Abstract

Policies at multiple levels pronounce the need to encompass both social and ecological systems in governance and management of natural capital in terms of resources and ecosystems. One approach to knowledge production and learning about landscapes as social–ecological systems is to compare multiple case studies consisting of large spaces and places. We first review the landscape concepts’ biophysical, anthropogenic, and intangible dimensions. Second, we exemplify how the different landscape concepts can be used to derive measurable variables for different sustainability indicators. Third, we review gradients in the three dimensions of the term landscape on the European continent, and propose to use them for the stratification of multiple case studies of social–ecological systems. We stress the benefits of the landscape concepts to measure sustainability, and how this can improve collaborative learning about development toward sustainability in social–ecological systems. Finally, analyses of multiple landscapes improve the understanding of context for governance and management.

**Electronic supplementary material:**

The online version of this article (doi:10.1007/s13280-012-0368-0) contains supplementary material, which is available to authorized users.

## Introduction

The global discourse about sustainable development (SD) as a societal process and sustainability as outcomes on the ground (WCED 1987; Norton 2005; Baker 2006) has been introduced in multiple policy areas including forestry, agriculture, energy, mining, and use of water as well as urban and rural development. However, translation of policy to practice remains a major challenge (Adger and Jordan 2009; Franklin and Blyton 2011). This applies to the extent to which different policy instruments and governance arrangements (Young 2013) are effective in different contexts, as well as what types of management deliver desired benefits (Puettmann et al. 2008). Moreover, there is increasing evidence that there are tipping points in both ecological and social systems (Angelstam et al. 2004a; Rockström et al. 2009; Villard and Jonsson 2009; Grimm and Schneider 2011) that cannot be passed without negative effects on sustainability outcomes or governance processes. Finally, climate change and global economics imply major uncertainties that stress the need for social learning toward adaptive management and governance of natural capital on which the human enterprise depends (Barnes 2006; Kumar 2010). The ecosystem services concept is an interface that aims at improving policy-makers’, governors’, planners’, and managers’ understanding about the benefits of ecosystems for society (Norgaard 2010; Potschin and Haines-Young 2012).

To produce knowledge and encourage learning that supports implementation of SD and sustainability policy, new modes of integrative problem-solving knowledge production have been proposed (Gibbons et al. 1994; Tress et al. 2006; Hirsch Hadorn et al. 2008; Axelsson et al. 2011). This stems from the need to understand the triad of ecological systems, social systems, and the behavior of the human being. Komiyama et al. (2011) used the terms global, social, and human systems to capture this triad. This diversity stresses the need for including in the knowledge production process both human and natural sciences (Snow 1993; Myrdal 2009), and learning by collaboration of academic and non-academic actors (Tress et al. 2006; Hirsch Hadorn et al. 2008; Angelstam et al. 2013a). Additionally, multiple spatial scales need to be covered, from points and patches to catchments, landscapes and regions (Forman 1995; Haggett 2001), time scales from diurnal fluctuations to long-term evolutionary changes (Delcourt and Delcourt 1988), and multiple levels of governance (Bache and Flinders 2004).

Contemporary policies about natural resources are often formulated to mitigate some kind of societal ill such as loss of particular species or landscape diversity, threats to the delivery of ecosystem services, or decline of cultural or esthetic values. This has triggered development of a diversity of scholarly terms that stress the notion of focusing on social–ecological systems (see Electronic Supplementary Material, Table S1). The term landscape, as used in different fields of geography, captures this and provides interfaces to a wide range of disciplinary approaches and knowledge.

Consequently, to translate the global discourse about SD and sustainability into action and desired outcomes on the ground, a wide range of policy documents advocate, in one way or another, an integrated landscape approach (e.g., WFC 2009; Axelsson et al. 2011, 2013b). This implies integration of governance and management in landscapes as spaces and places. The landscape approach addresses the desire in policies and among scholars to include both social and ecological systems in research and development, thus implicitly stressing the need to carry out inter- and transdisciplinary research (Wu 2006; Naveh 2007; Wu and Hobbs 2007).

Capturing ecological systems, social systems, and the behavior of the human being in a holistic manner requires common frameworks (Ostrom 2009) to compile and synthesize knowledge. We argue in favor of using multiple landscapes, that is spaces and places, as case studies (see Flyvbjerg 2011; Gill 2011) for comparative studies about SD and sustainability (see also Liu et al. 2007; Potschin and Haines-Young 2012). This is consistent with the terms natural experiment (sensu Diamond 1986) or labscape (Kohler 2002), but also comparative politics (Landman 2003). As a start, to allow for meaningful comparative studies, multiple landscape case studies need to be stratified based on the different dimensions of landscape concepts. For a given biophysical context, Angelstam and Törnblom (2004) proposed stratification of multiple social–ecological systems as case studies with respect to landscape histories, which affects the state of different sustainability dimensions, and to systems of governance, which affect the way society is steered.

The European continent hosts a diversity of natural biophysical conditions, economic histories and thus the tangible legacies of impacts on social–ecological systems. Intangible conditions such as levels of economic backwardness and bureaucratic rigidity, as well as cultures of politics and governance arrangements (Gunst 1989; Janos 1989; Davies 1997; Katchanovski 2006) are also diverse. Such gradients, when steep enough, are even termed fault lines (Bugajski and Pollack 1989; Huntington 1997). To implement policies about SD and sustainability in European landscapes thus requires regionally and temporally adapted solutions. There is also great opportunity for innovative knowledge production based on comparisons of multiple landscapes as case study areas in different regions of the European continent (Angelstam et al. 2011a, 2013c, d).

The aim of this paper is to present the different landscape concepts as an interface to both human and natural science knowledge production, as a practical tool for social learning on the ground, and to design and carry out multiple case studies for comparative transdisciplinary research of social–ecological systems as large spaces and places. First, we review the landscape concepts’ natural, anthropocentric, and intangible interpretations as defined in the wide range of landscape research schools that have emerged, especially in Europe’s East and the West. Second, we exemplify how the landscape concepts can be used to derive measurable variables for sustainability indicators. Third, we use the European continent to illustrate the main gradients that need to be considered to achieve variation in different landscape dimensions when carrying out comparative landscape case studies related to SD and sustainability among countries and regions. Finally, we discuss the usefulness of the landscape concepts for supporting knowledge production about landscapes by measurement of sustainability indicators, collaborative learning at multiple levels from local to national, and international networking for transdisciplinary research about SD and sustainability.

## The Diversity of Landscape Concepts

### Multiple Interpretations and Scales

The word landscape occupies a broad niche in human culture. Covering such different fields as geography, ecology, arts, and philosophy, landscape has various interpretations, and there have been several approaches to classify or systemize them (e.g., Meinig 1979; Armand 1975, 1988; Jones 1991; Grodzynskyi 2005). Landscape is also spatially explicit, and encompasses a wide range of spatial and temporal scales (Liu and Taylor 2002). Finally, it encompasses methods to identify and measure themes or information layers that include both tangible and non-tangible values (Head 2004; Axelsson et al. 2013a).

The typology of landscape interpretations proposed (Table 1) is designed for enhancing a transdisciplinary approach to knowledge production and learning for SD toward ecological, economic, and social sustainability. These three pillars are also parts of different landscape interpretations. We divide landscape concepts into four groups; first three more narrow concepts, namely biophysical or natural, anthropogenic, intangible, and then one that merges them to one.

**Table 1:** Typology of four landscape concepts and their interpretations as sub-groups

Index	Type of interpretation	Fields where it is most commonly used
*Biophysical interpretations*		
Landscape as purely natural phenomenon		
BPh-1	Territorial complex composed of the natural components (rocks, soils, vegetation, etc.)	Traditional Soviet Landscape Science
BPh-2	Area organized in a system by biophysical patterns and processes	Landscape Ecology
BPh-3	Area preserved in its pristine natural image (wilderness and naturalness)	Layman’s interpretation
*Anthropogenic interpretations*		
Landscape as nature with human artifacts		
Ant-1	Spatial system composed of natural and anthropogenic elements	German Landschaftskunde; Landscape Ecology
Ant-2	Space with specific interactions between human culture and natural environment	Cultural Geography, French Geographie humaine
Ant-3	An area physically perceived as spatial integrity	Common people’s interpretation, policy documents
*Intangible interpretations*		
Landscape as cognitive representation of a space, socio-economic interpretations and landscape as socially organized space		
Int-1	Visual image of an area	Common people’s interpretation, Perceptual Geography
Int-2	Mental image of a space	Psychology
Int-3	Landscape as composition of places bearing moral and ethical values	Humanistic Geography, Phenomenology
Int-4	Landscape as an area specific with its economical and social functions	Spatial planning
Int-5	Landscape as place for humans, arena where their behavior is taking place	Behavioral geography
Int-6	Landscape as esthetically organized space, an area giving esthetic satisfaction	Landscape design; Environmental aesthetics
*Coupled social*–*ecological interpretation*		
Landscape as totality including both material natural and cultural dimensions, and spiritual phenomena (see also SM Table 1).		
CSE	Total system including both tangible and intangible elements	French Geographie humain; Geosynergetics of J. Schmithüsen, Space–time Geography of Hägerstrand, “Total Human Ecosystem” of Naveh

First, the biophysical landscape concept consists only of biophysical elements (e.g., topography, bedrocks and soils, vegetation), and excludes anthropogenic elements like buildings, roads, and even agricultural fields. The traditional Soviet school of landscape science’ interpretation is a good example (Solntsev 1948, 1962; Isachenko 1991; Dyakonov et al. 2007). Second, the anthropogenic landscape concept sensu Milkov (1973) adds anthropogenic elements to the biophysical landscape, but does not consider intangible elements like human beliefs, ethical norms, and other values as a part of it. Third, another concept insists that intangible values are as important as tangible biophysical natural ones and anthropogenic elements (Bobek and Schmithüsen 1949; Naveh 2007). Thus, subjective representation of a landscape in the human mind [human geography sensu Seamon (1984) and Cosgrove (1993)] and environmental psychology (Altman and Rogoff 1987) are included into this landscape dimension. Land property, income, and class are examples of other intangible elements of landscapes. This triad has been noted by a wide range of scholars including Sauer (1925), and Bobek and Schmithüsen (1949) who used the terms natural, cultural, and subjective (Geistlich in German). Fourth, the integrated interpretation of landscape combines these three landscape concepts, viewing landscape as a totality (Hägerstrand 1985). These concepts of landscape in science and humanities, and by lay persons, can also be divided into several more specified sub-groups of interpretations (Table 1).

The pattern, configuration, and spatially explicit features behind processes in landscapes mean that the problem of spatial scale is crucial for studying landscapes (Allen and Hoekstra 1992; Wiens 2005). While landscape ecology is mostly interested in landscapes’ spatial and, to a lesser extent, temporal scales, its applications to integrated management and sustainability issues also requires the social scale to be considered (Hansson and Angelstam 1991; Field et al. 2003). Generally speaking, the landscape concepts may work at various levels of space, time and of social life, but their efficiency on these levels is not the same. The landscape concepts work especially well if it is applied for areas of 1 ha to 10 000 km^2^ in size, for a time frame of 1–100 years, and in the social scale from local to regional communities.

### Biophysical Interpretations

Biophysical interpretations of the term landscape emphasize that a landscape is above all a natural phenomenon, which evolved by natural biophysical processes and is still by and large controlled by these. There are at least four modifications of this interpretation (Table 1). The first and most developed biophysical interpretation is the ‘natural terrain complex’ (NTC) (BPh-1 in Table 1). The term landscape (landshaft in Russian) was apparently borrowed from German geography (Berg 1915). This interpretation was developed within the Soviet landscape science school (Landshaftovedeniye in Russian), and later within the theory of geosystems (Ucheniye o Geosistemakh in Russian) by Sochava (1978), which have also influenced national landscape schools of contemporary Eastern Europe. The NTC interpretation was originally strongly supported by the prevailing and obligatory philosophical Marxist paradigm in the USSR that demanded objective reality in nature. According to the proponents of the landscape as a NTC, a landscape is a natural unit where the components of the natural environment (rocks, soils, climate, flora, fauna, etc.) have a high degree of interdependence, which creates spatial patterns of distinct character. Any products of human activities, even if they are physically present within the landscape, are not included to this interpretation (Solntsev 1962; Isachenko 1991). The resulting landscape maps thus do not show the actual landscapes, but the landscapes that theoretically or potentially should be without human interferences, neither in the past nor at the present (Troll 1950; Annenskaya et al. 1965). The spatial flows of matter in catchments and between landscape units is the core of the geochemical landscape interpretation (Kasimov and Gennadiev 2007), which has been applied effectively for pollution assessment, agricultural planning, and mineral exploration. NTC maps are still applied widely for various practical issues including land assessment and management, monitoring, and spatial planning (Dyakonov et al. 2007). Another example of the use of the biophysical landscape interpretation BPh-1 for SD issues is “nature potentials” first presented by Neef (1966, 1967), and then developed by his followers from the Dresden-Leipzig landscape school (Mannsfeld 1979). The landscape’s natural potential is an informative indicator of the sustainable use of natural resources and ecosystem services.

The second interpretation is represented by the various forms of landscape ecology linking pattern and process (BPh-2 in Table 1; see Turner et al. 2001; Turner 2005). In Europe this developed from Troll’s (1950) interpretations of air photos, and was later inspired by island biogeography and dispersal ecology, but transferred to anthropogenic landscapes mainly due to the marked technological and structural changes in European agriculture. Here the core of the term landscape lies in spatial flows, most often biotic migrations, and organizing land units into distinct natural systems. The spatial structure of a landscape is thus interpreted as a pattern of patches of natural ecosystems connected with each other by the routes providing corridors for species migration (Forman 1995). Although in this model of landscape the human factor is present, it is considered as a matrix (i.e., any area not covered with natural and semi-natural vegetation) upon which the true essence of the landscape (its biotic life, migrations, survival, extinctions and the like) is concentrated. This interpretation (BPh-2), unlike BPh-1, emerged and is developing successfully in North America and West Europe. It is especially effective in wildlife management and biodiversity conservation (e.g., Hansson and Angelstam 1991), and provides a scientific background for planning and management of habitat networks (Nowicki et al. 1996; Andersson et al. 2012b). In the former USSR states, and especially in Russia where natural landscapes are still dominating on its vast areas, the landscape matrix idea is not as popular as in the West, and is used only occasionally in planning of ecological networks (Deodatus and Protsenko 2010). Landscape ecology continues to evolve by becoming more anthropocentric. For example, Haines-Young (2000) recognized the need to understanding the limits for ecological functions that are important for people. Stressing that humans, as any species, are a part of ecological systems, social landscape analysis draws upon theoretical foundations in applied demography, human ecology, and rural community studies (Field et al. 2003).

The third biophysical interpretation is naturalness (BPh-3 in Table 1), meaning that only areas where natural environment remain untouched by humans are regarded as landscapes, while the rest are not. For solving ecological sustainability issues in human-modified European landscapes, the BPh-3 interpretation is a good reference point to study the degree of a landscape’s naturalness (sensu Peterken 1996). Many terms are used to describe the conditions in naturally dynamic ecosystems, such as ecological integrity (Pimentel et al. 2000), resilience (Gunderson and Holling 2002), historic range of variation (Egan and Howell 2001), hemeroby and naturalness (Egan and Howell 2001). According to Peterken (1996) the degree of naturalness describes the gradual loss of composition, structure and function of ecosystems with increasing human alteration [see also Angelstam and Dönz-Breuss (2004), and Brumelis et al. (2011) who used the analogous terms species, habitat and process]. The development of naturalness is linked to the type of ecosystem and its disturbance regime. For example, in the boreal forest biome, where disturbance intensity and frequency can be high, forest naturalness includes not only old-growth stands but also recent burns and windfall areas (Angelstam and Kuuluvainen 2004). Therefore, the degree of a landscape’s naturalness, while being adapted to local and regional specificities of landscape history, is a valuable ecological indicator of sustainability (Electronic Supplementary Material, Table S2).

### Anthropogenic Interpretations

The anthropogenic landscape concept focuses on material products of human activities in a landscape. There are several interpretations of this, each stressing a particular type of anthropogenic element or type of relations with the natural environment (Table 1). The landscape interpretation Ant-1 is widely used, and stresses that a landscape is a part of space where the natural elements, and those introduced or modified by humans, are closely interrelated, thus creating integrity with distinct character, as well as social and ecological functions. This interpretation originated in the German Landschaftskunde (Schluter 1920), was then developed in the anthropogenic landscape science by Milkov (1973), and adopted in European landscape ecology, which uses it extensively for landscape planning, land and resource management (e.g., Zonneveld 1995; Richling and Solon 1996). Although the BPh-1 interpretation gained prevailing support for describing the most relatively undisturbed area in Russia, it is not surprising that Milkov’s interpretation, being in line with the Ant-1 interpretation, emerged in the Voronezh scientific school, that is in totally transformed steppe and forest-steppe region in today’s Russian Federation. The anthropogenic interpretations of landscape provide a theoretical platform for analyses of multiple features of managed landscapes. Additionally, the spatial correlation between land-use pattern and pattern of natural landscape features can be used to indicate the level of discrepancy between natural and human-imposed landscape heterogeneity.

While landscape interpretation Ant-1 is more European, placing special emphasis on economic utilization, transformations and optimization of landscapes, another interpretation of the term landscape as an anthropogenic category was developed in the USA under the title of cultural landscape. It was elaborated by Sauer (1925), who stated that the cultural landscape emerged from the natural landscape as a result of it being shaped to human needs by local practices and cultural traditions. Paying special attention to cultural traditions and human interactions with the natural environment Sauer (1925) asserted that the cultural landscape is above all a biophysical entity and considered human culture as its factors. Thus, while interpretation Ant-1 pays particular attention to anthropogenic elements of a landscape related primarily to economics, interpretation Ant-2 focuses on landscapes’ cultural features. However, Farina (2000) extended this to include also economic dimensions. Social and cultural sustainability indicators may be constructed on the basis of both (Table S2).

In contrast to the anthropogenic landscape interpretations Ant-1 and Ant-2, which have strong scientific backgrounds, the interpretation that the landscape is the area physically perceived as having spatial integrity (Ant-3) is more intuitive and subjective. It is about how the term landscape is often understood by common people. This interpretation opens up for flexible operation using the term landscape. It also explains why interpretation Ant-3 is used in some political documents, including the European Landscape Convention where the landscape is defined as “a zone or area as perceived by local people or visitors, where the visual features and characteristics of the landscape are the result of the action of natural and/or cultural factors” (Anon. 2000).

The advantage of anthropogenic interpretations of the term landscape lies in presentation of landscapes as biophysical nature–anthropogenic entities. In particular, they could be ranked along an axis from more or less anthropogenically transformed; the historic approach could be used for tracking and predicting changes of pattern and functional composition of anthropogenic land landscapes and assess landscape functions (e.g., Bastian 1999). At the same time, the anthropogenic interpretations of landscape remain mainly biophysical. In their attempt to explain nature–culture and nature–economy interrelations in a landscape, they generally do not explicitly regard other intangible social and cultural elements as intrinsic parts of landscapes.

### Intangible Interpretations

Intangible interpretations of landscapes include cognitive and perceptual aspects of the landscape, stressing that the landscape is not a material entity of the physical world but its representation in human mind (Entrikin 1991; Cosgrove 1993). Depending on the form of this representation (e.g., visual image, mental image, text, metaphor) various interpretations of the term landscape have been proposed.

The simplest and the earliest is the interpretation Int-1 (see Table 1) of landscape as a visual image of an area. Any person has a personal perception the landscape (Bailly et al. 1980). The Int-2 interpretation is broader and deeper than that of Int-1, because the landscape is perceived not only visually, but in many other perceptual and cognitive forms, including attaching various meanings and values to it (Seamon 1984). Taken together, they create a multidimensional image of a space in humans’ minds. The Int-2 interpretation of a landscape is used mostly in psychology, whereas for geographical sciences the more spatial mental interpretation Int-3 is used. According to this interpretation, human individuals and communities attach some meanings and values to different places. In the human mind these places are connected to each other by particular meanings, associations, reminiscences, and feelings creating entities called landscapes. They are spatial and patterned, not in the physical space, but in the human brain. Thus, the landscape interpretation Int-3 is the perception of an organized and meaningful part of space.

Although the cognitive interpretations of landscape were not intentionally designed for any practical purposes, landscape phenomenological interpretations as topophilia (Tuan 1974), placelessness (Relph 1976), and biophilia (Wilson 1984) have numerous applications in city planning and restoration of cultural landscapes (Relph 1981; Porteous 1996). There are also other cognitive interpretations of landscape in poetry, visual arts, and in other fields of humanities (Appleton 1990; Grodzynskyi 2005). Being of interest to many SD and sustainability issues, they can hardly be used solely, but could be nicely coupled with other landscape interpretations.

Another suite of intangible landscape interpretations are socio-economic, and stress landscapes’ importance for humans. At least three interpretations could be mentioned (Table 1). First, landscape is interpreted from an economic standpoint as the area spatially differentiated into its parts each performing particular economical and social functions (Int-4) (Krugman 1994; Oueslati and Salanie 2011). Similarly, power and legal rights are not physically manifested but are crucial to stakeholders’ sense of place. In many cases the spatial structure of landscape is interpreted as a pattern of land uses or as its functional zones, such as agricultural, recreational, or protective. The core of the economic interpretation of a landscape is to analyze how social and economic activities, property, social class stratification, and income are distributed around a particular part of a space. These are core topics of economic geography and regional science. Second, behavioral geography (Int-5) proposes landscape as an arena where human behavior is taking place (Barker 1968). A landscape’s spatial pattern is often interpreted as the configuration of behavioral places where human life is organized (Golledge and Stimson 1997). The idea of behavioral landscape has been explored in various themes including city and spatial planning, and exploration of places preferred by humans (Walmsley and Lewis 1993). The geographical division of property, income, classes and ethnicity between and within areas are also important. Third, esthetic interpretations of landscape (Int-6) see landscapes as designed by humans in order to satisfy their esthetic demands (Appleton 1975; Bourassa 1991). This is used for landscape beauty evaluation, city planning, recreation, and management of rural and other areas (Zube et al. 1982; Grodzynskyi and Savytska 2005). Landscape preference criteria (Kaplan and Kaplan 1989) could be borrowed from intangible interpretations of landscape and used as indicator variables of SD (Table S2).

### Coupled Social–Ecological Interpretations

The review of the existing three groups of interpretations of the term landscape shows their variety and differences, which at first glance seem incompatible. This has made scholars make pleas for unified landscape concepts, thus moving different landscape schools closer together and collapsing the distinctions among them (Head 2004; Huggett and Perkins 2004; Wiens 2005; Potschin and Haines-Young 2006). In addition to viewing landscapes as mainly biophysical, anthropogenic, or intangible, scholars thus advocate the concept of landscape as totality (Antrop 2004; Naveh 2007). Nevertheless, the landscape concepts’ biophysical, anthropogenic, and intangible dimensions of the coupled socio-ecological concept, which integrates all of them, provide interfaces to both human and natural science disciplines (sensu Snow 1993), and thus to theoretical frameworks that can be used to describe global, social, and human systems (sensu Komiyama et al. 2011). This forms an important foundation for deriving measurable variables for ecological, economic, social, and cultural sustainability indicators. In Table S2 (see Electronic Supplementary Material) we have compiled a suite of examples of such variables, which illustrate how different landscape interpretations can contribute to the measurement of different aspects of sustainability.

Rooted in the French ‘geographie humaine’ with its primary concern of landscapes as the spatial nature-societal-cultural-historic entities specific with their ‘genre de vie’ (Vidal 1911), the idea of landscape totality is not new. The tradition of ‘geographie humaine’ is now used most successfully also in Romania and Finland. For example, the Finnish geographer Keisteri (1999) studied landscapes as entities characterized by a specific lifestyle, indicated on signs and other landscape elements, and which are essential both to preserving local identity and to human everyday life. In the context of modern holistic interpretation of landscape the works of Hägerstrand (1985) should also be mentioned. Responding to an urgent need in integrative approaches he provided holistic social–ecological interpretation of spatial systems. Also landscape ecology has partly evolved to claim that landscape as totality provides a platform for interdisciplinary studies, embracing natural biophysical, anthropogenic, and intangible elements into one holistic system (e.g., Wu 2006).

Material and intangible elements are closely interrelated and influence each other. Scholars have argued that it is incorrect to consider them separately (Bobek and Schmithüsen 1949; Claval 2004; Naveh 2007). The landscape concepts are hence not owned by any particular discipline or school. As stated by Head (2000), landscape “is a concept whose problematic status makes in interesting”.

The broad understanding of the term landscape has its pros and cons. Its strong advantage lies in the field of general methodology as its interpretation enhances comprehensive analysis of an area or of a complex problem incorporating the variables, scales, and proper theories to be employed. We thus view the landscape concepts and their different interpretations as a proper basis for inter- and transdisciplinary knowledge production and learning. However, in cases of small projects, or while solving particular narrow issues of the landscape, it could be redundant, and therefore the concept is unlikely to be informative as to how the landscape should be analyzed, mapped, protected, and, finally, managed. This is perhaps the only limitation of the interpretation of landscape as totality. As any other concept or interpretation it has its domain. Despite being broad, the concept of landscape as totality does not replace three other ‘partial’ interpretations of landscape. We argue that they may be exploited effectively for deriving measurable variables about landscapes (see Table S2), and then integrated into broader landscape picture on the platform of coupled social–ecological interpretation of a landscape.

## European Gradients for Stratification

**Fig. 1:**
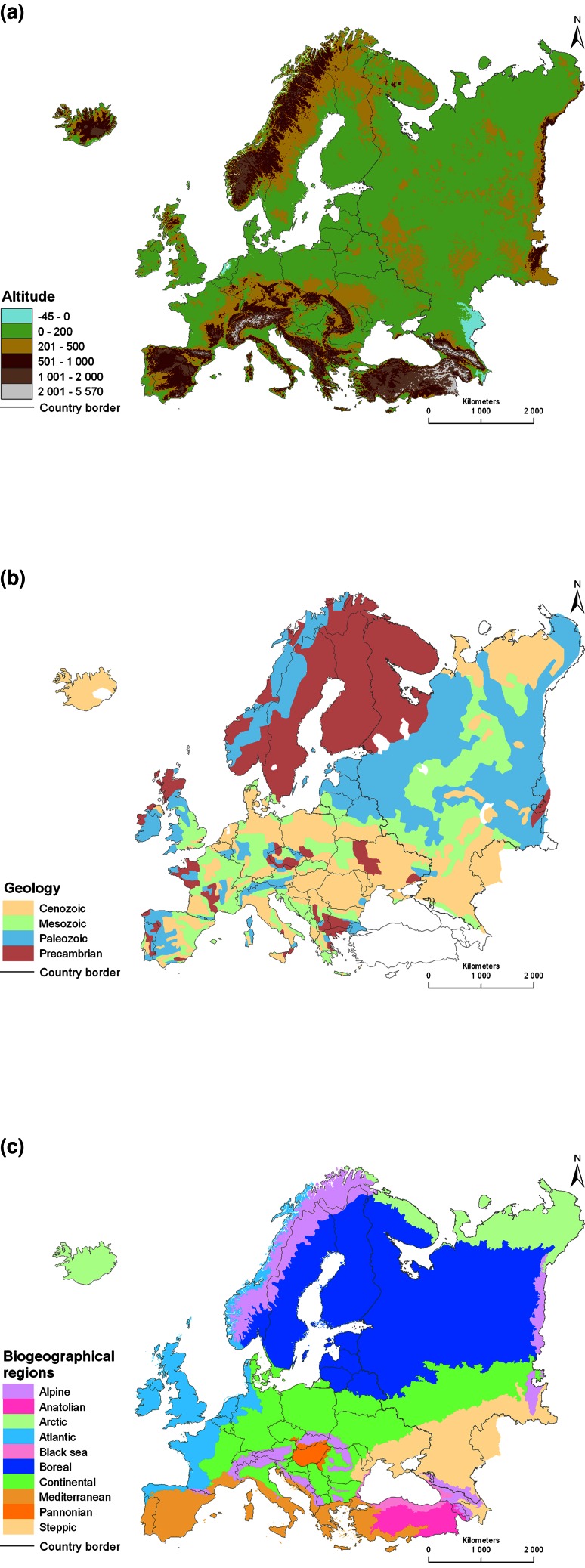

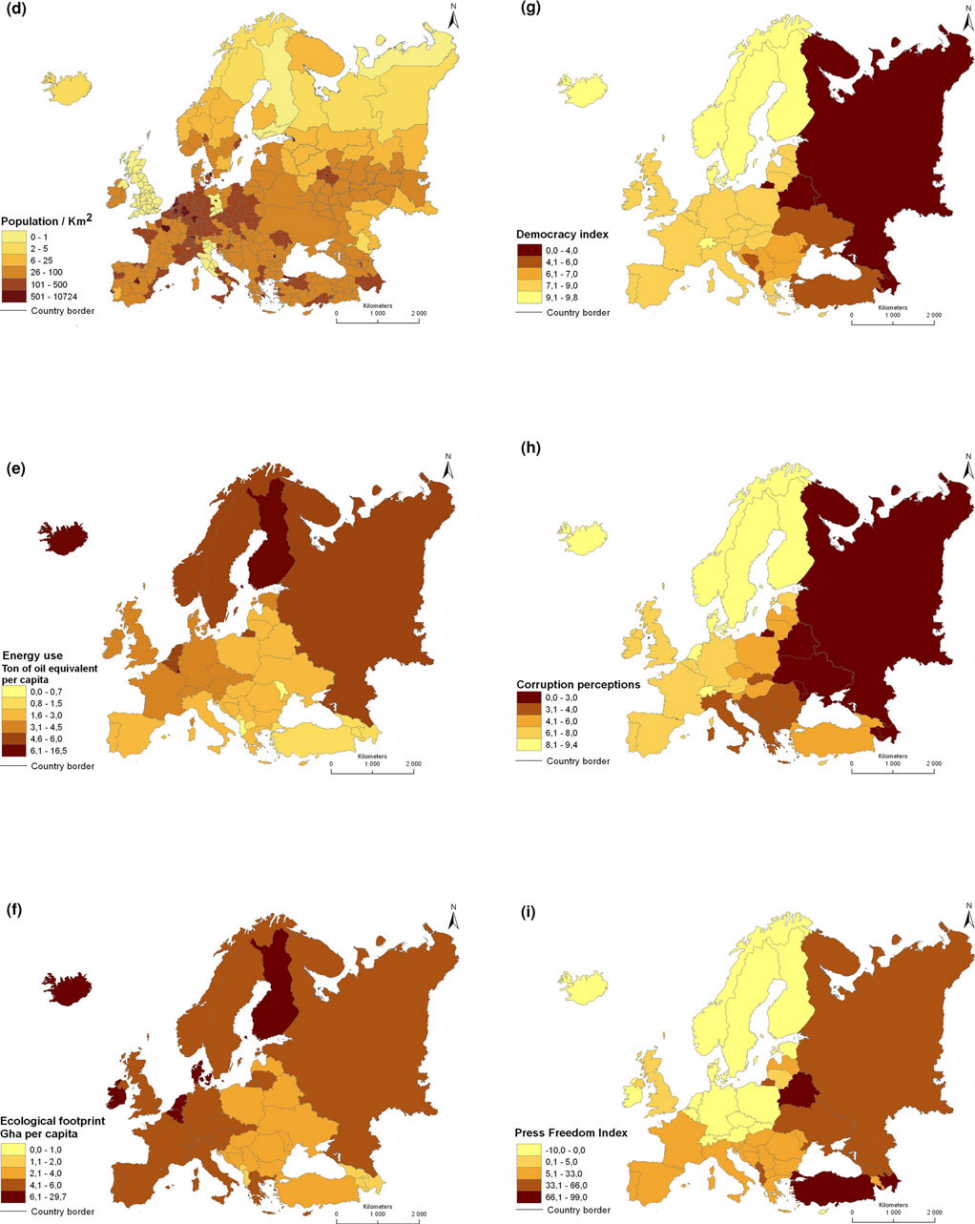
Maps of biophysical (**a**–**c**), anthropogenic (**d**–**f**) and intangible (**g**–**i**) landscape dimensions in Europe. **a** Altitude in relation to sea level (Available online at http://eros.usgs.gov/#/Find_Data/Products_and_Data_Available/GTOPO30; retrieved 8 August 2012). **b** Geology in terms of formations and deposits (Generalized based on http://www.geolocation.ws/v/W/File:Europe%20geological%20map-en.jpg/-/en; retrieved 8 August 2012). **c** Biogeographical regions in Europe (official delineations used in the EU Habitat Directive (92/43/EEC) and for the EMERALD Network under the Bern Convention) (See http://www.eea.europa.eu/data-and-maps/figures/biogeographical-regions-europe-2001/biogeo_graphic.eps; retrieved 23 August 2012). **d** Population density by European Union NUTS 2 regions, Belarus, Ukraine, Moldova, Serbia, Bosnia and Herzegovina, Georgia, Armenia and Azerbaijan (Data from http://data.worldbank.org/indicator/EN.POP.DNST), subjects of the Russian Federation (Data from European Commission Eurostat http://epp.eurostat.ec.europa.eu/statistics_explained/index.php/Population_change_at_regional_level, retrieved 23 August 2012; and Federal State Statistics Service 2010). **e** Energy consumption in terms of 1000 kg oil equivalent per capita (Data online from http://data.worldbank.org/indicator/EG.USE.PCAP.KG.OE, retrieved 24 October 2012). **f** Ecological footprint 2008 by countries (Global Footprint Network 2011). **g** Democracy index (Economist Intelligence Unit 2011). **h** Corruption perceptions index (Transparency international 2011). **i** World Press Freedom Index 2011–2012 (Reporters without borders 2012)

Any research design aimed at studying relationships among different variables is based on replicated data collection in situations that represent sufficient variation in the variables of interest, and with a sufficient sample size (e.g., Landman 2003). To obtain a holistic understanding of ecological, economic, social, and cultural consequences of the ways natural resources are used and managed, and products produced, it is necessary to have data points that represent entire social–ecological systems, or landscapes. At the same time, individual case studies provide depth (Flyvbjerg 2011; e.g. Richnau et al. 2013). Focusing on stratification of countries and regions for selection of social–ecological systems as case studies (Angelstam et al. 2013c, d), the European continent is a very diverse peninsula in the westernmost part of the Eurasian land mass, and has many steep gradients (e.g., Davies 1997). To illustrate this, the spatial pattern of biophysical, anthropogenic, and intangible landscape dimensions (Table 1) on the European continent was illustrated with data from 53 countries. These included all the 27 EU Member States, its candidate countries, as well as Switzerland, Norway, Turkey, Belarus, Ukraine, Georgia, Armenia and Azerbaijan, and the Russian Federation west of the Ural Mountains (Fig. 1).

Regarding biophysical gradients, altitude (Fig. 1a) and geology (Fig. 1b) are key determinants of topography. Together with the climate they determine the location of different biogeographical regions (Metzger et al. 2005; Jongman et al. 2006) (Fig. 1c). Regarding anthropogenic gradients, examples of indicators include human population density (Fig. 1d), energy consumption (Fig. 1e), and ecological footprint (Fig. 1f), all of which form proxies for reduced levels of naturalness. Finally, examples of intangible landscape dimensions are linked to political culture and thus governance include indicators of democracy (Fig. 1g), freedom of press (Fig. 1h), and perceived corruption (Fig. 1i).

Two gradients, viz. landscape history and governance arrangements, are important stratification variables from the point of view of Europe as a laboratory for selecting multiple spaces and places as case studies as natural experiments for transdisciplinary research about sustainable natural resource management (e.g., Angelstam et al. 2011a). This applies to any large biophysical unit’s natural resources, such as forest and woodland in the boreal and temperate ecoregions (Fig. 1c).

**Fig. 2:**
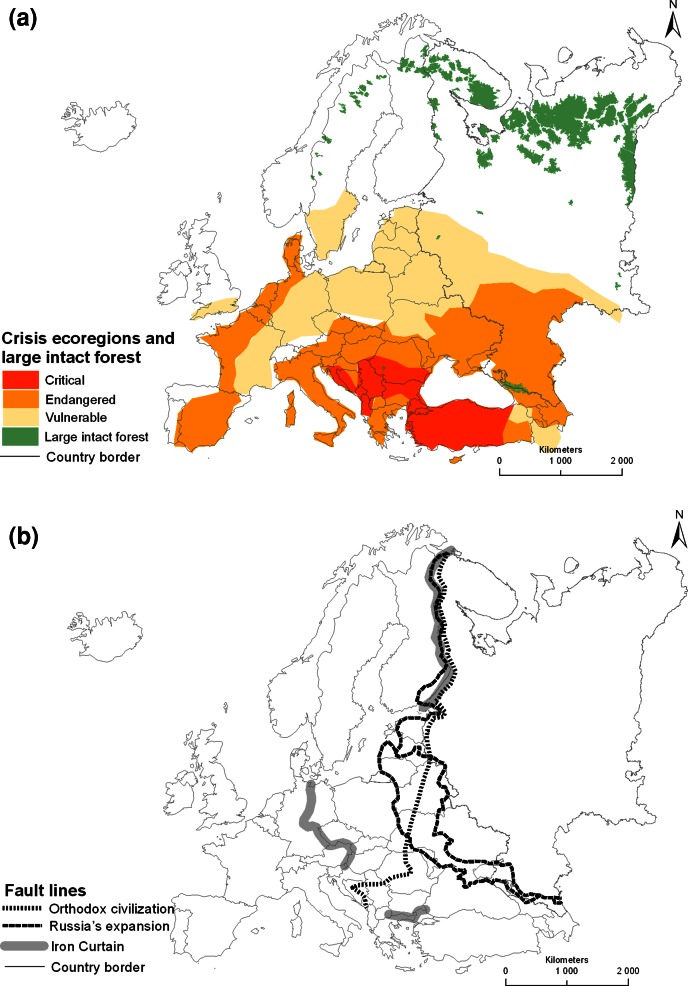
Map of Europe as a laboratory for selecting multiple social–ecological systems as case studies, and thus natural experiments (sensu Diamond 1986) with two gradients as key stratification variables. The first, landscape history, is indicated in (**a**). This shows the north–south gradient in the level naturalness of landscapes, from large intact forest landscapes (Potapov et al. 2008) to Hoekstra et al.’s (2005) identification of crisis ecoregions as vulnerable, endangered, and critically endangered. The second, generally oriented west–east (**b**), is linked to European fault lines of governance and political culture in the wide zone from the Iron Curtain in the west, separating countries linked to NATO and the former Warsaw pact (Niblett and Wallace 2001), the western expansion of Russia during the reign of Catherine II 1772–1795 (Skinner 2009), and associated western boundary of the orthodox civilization (see Wallace 1990; Huntington 1997; Skinner 2009)

The first gradient, generally south–north on the European continent, is landscape history linked to the gradual expansion of the human enterprise and its effects on ecosystems as natural capital (Angelstam et al. 2013b). Commonly, countries are used as units of study of economic development (Rostow 1960; Landman 2003), which is a major driver of landscape change. However, in addition, the regional level can contribute with improved spatial resolution. A good example is Central Europe where the level of economic backwardness was linked to the historic expansion and contraction of Germany, Russia as well as the Habsburg and Ottoman empires (Gunst 1989; Sylla and Toniolo 1991; Hanioglu 2008). Regarding the ecological system, human conversion of natural habitat is the largest single cause for loss of biological diversity, that is composition, structure, and function of ecosystems. Europe’s Mediterranean south and boreal north forms a clear gradient in the loss of habitat (Hannah et al. 1995) and level of ecoregional vulnerability (Hoekstra et al. 2005). Conversely, large intact forest landscapes remain only in remote northern regions (Fig. 2a). To conclude, the clearing of natural ecosystems for agriculture which began in the eastern Mediterranean ecoregion several millennia BP, and much more recently of boreal timber frontiers during the past century have spread from centers to peripheries of economic development (sensu Gunst 1989).

The second gradient, generally west-east oriented, is linked to regional differences in European history (Berend 1986; Best 2009), political culture (Katchanovski 2006) and religion (Wallace 1990; Davies 1997; Skinner 2009). This gradient is particularly steep in the zone from western border of Russia at the end of the eighteenth century in the east to the western border of the Warsaw pact (Fig. 2b). Countries west of this zone, within this zone, and further to the east, exhibit distinct differences in democracy, perceived corruption, and freedom of press (Fig. 1g–i), all of which are linked to the systems of governance. Concerning the social system human geographers and historians have in fact for long time attempted to develop world views of geopolitical relationships (Mackinder 1904; Spykman 1944; Cohen 1964; Blake et al. 1987; Niblett and Wallace 2001). The European continent is indeed a good example of a gradient in political culture (e.g., Katchanovski 2006). Focusing on Europe as geographical unit from the Atlantic Ocean to the Ural Mountains, Huntington (1997) proposed that there are two civilizations—the Western and the Orthodox. The cultural fault line, or rather wide zone (see Fig. 2b), between the two is closely associated with the EU’s expansion to the east, and runs along the western border of the Russian Federation, divides Belarus, Ukraine, and Romania into different spheres of influence, and separates Slovenia and Croatia from the rest of the former Yugoslavia (Fig. 2b). To conclude, there are three strata linked to societal steering (Katchanovski 2006); viz. (1) western civilization sensu Huntington (1997) west of the former Warsaw pact, (2) countries in transition, and (3) orthodox civilization sensu Huntington (1997) east of the western border of the Orthodox religion.

## Discussion

### Landscape Concepts as Tools to Measure Sustainability

SD and sustainability are often viewed as confusing and complex concepts (see review by Dresner 2008). While the first focuses on the societal process, the second focuses on what this process results in. In this paper we argue that the multi-faceted interpretations of the term landscape provide an interface to a wide range of disciplines. Broadly speaking there are three different landscape concepts that focus on different aspects of landscape, and a fourth that integrates all the three. From the perspectives of knowledge production for sustainable use of natural resources, all landscape schools have their advantages in terms of methods for providing systematic description of spaces and places.

Our review of landscape concepts and their interpretations also demonstrate the importance of context to understand why there is different focus on different landscape concepts in time and space. For example, the biophysical interpretations of landscape, which dominated in the former USSR and Eastern Europe, have their pros and cons. The shortcomings of the biophysical landscape interpretations come from unrealistic assumption that the landscape is only a natural phenomenon. Later there were attempts to widen the notion of landscape by including cultural phenomena. These ideas, however, were not supported by the Eastern European scientific community, which at that time was under the communist control and did not recognize the importance of interconnections between the natural environment and the societal development. Since the 1990s after the collapse of former ideological limitations, the concept of cultural landscape is developing rapidly in Russia (Kalutskov 2007). The trend toward Alexander von Humboldt’s concise definition of landscape as “der Totalcharakter einer Erdgegend” (Zonneveld 1995) also occurs in the West. For example, as exemplified by Wu (2006) and Musacchio (2009), there is an emerging wide-spread argumentation in favor of the diversity of landscape concepts as tool for sustainability science and a human-centered perspective (e.g., Field et al. 2003; Kates 2011).

To conclude, we propose that the different landscape concepts and their interpretations can be used as a foundation to combine a suite of human and natural science theoretical frameworks that allow measuring different aspects of landscapes with a holistic perspective. While this satisfies the knowledge production part of transdisciplinary research by identifying measurable variable for different pillars of sustainability, it needs to be complemented by social learning on the ground to make the knowledge useful in practice (e.g., Kates 2011).

### Landscape as Space and Place for Collaborative Learning

The production of new knowledge is characterized by both the new knowledge itself and the ways in which this new knowledge is learned and used (Gibbons et al. 1994). Learning based on knowledge about the state and trends of sustainability in a local landscape or region is enhanced if the stakeholder group includes different sectors and levels, different interests, and if the participants have different experiences and backgrounds (Brulin and Svensson 2012). This process of learning in a local landscape is complex, and requires that people with different skills contribute, and that stakeholders are open-minded and willing to participate in the learning process. In addition, a collaborative learning process often benefits from facilitation (Daniels and Walker 2001). To encourage learning for sustainable landscapes on the ground using a landscape approach (e.g., Axelsson et al. 2011), the challenge is to proceed from experiences to learning while generating knowledge in steps. A first step includes the local level process, where projects develop solutions to different problems, or particular sectors practice governance and management resulting in local experiences (e.g., Axelsson et al. 2013b). A second step involves learning from these local experiences, and to improve practices locally. A third step is to contribute to general learning based on local experiences and knowledge production, i.e., to go from tacit to explicit knowledge (Nonaka and Konno 1998). Systematic collection of stratified information from case studies provides relevant context-dependent knowledge that can be used in practice (Andersson et al. 2012a; Elbakidze 2013a).

This kind of multi-stakeholder learning process could be termed collaborative learning (Daniels and Walker 2001; Gray 2008). It takes place when project results are assessed, when stakeholders learn about each other, try to understand why a solution worked, what kind of problems there were, where it could have failed and relates it to their own experiences, i.e., to reflect on projects and the results (Svensson et al. 2009). When these prerequisites are met the result can be the creation of a space for learning (Nowotny et al. 2001). Collaborative learning processes will benefit from analyses of the involved stakeholders’ interests (Daniels and Walker 2001; Svensson et al. 2009), input of needed knowledge and the comparison of results with theories (Svensson et al. 2002). However, ‘socially robust solutions’ may simply mean solutions that do not affect the power relations among stakeholders. Transparent knowledge about the state and trends of sustainability at multiple levels, and systems analysis (e.g., Hjorth and Bagheri 2006) is empowering, and can thus support handling the relation between changes toward sustainability and related changes in power relations.

### Multiple Landscapes for Transdisciplinary Research

The use of a transdisciplinary approach includes identification of problems and challenges to produce new knowledge and to use collaborative learning to produce socially robust solutions (Nowotny et al. 2001; Svensson et al. 2009). We argue that there is great opportunity for innovative knowledge production about both governance and management for different landscape dimensions based on comparisons among multiple landscapes. As pointed out by Liu et al. (2007) integrated studies of social–ecological systems, or landscapes, reveal new and complex patterns and processes that are not evident when studied by social or natural scientists separately. Their studies of multiple social–ecological systems as case studies show that couplings between human and natural systems vary across space, time, and organizational units. Social–ecological systems, or landscapes, also exhibit nonlinear dynamics with thresholds, complex feedback loops, time lags, resilience, heterogeneity, and surprises. Additionally, there are legacies of the past that have effects on present conditions and future possibilities (Angelstam et al. 2011a, 2013b). However, in addition to Liu et al.’s (2007) example of interdisciplinary research, in order to contribute to the solution of problems related to the governance and management of natural capital, stakeholders and actors need to develop knowledge production and learning together. Transdisciplinary research captures this (e.g., Hirsch Hadorn et al. 2008). To increase the opportunity to generalize from multiple case studies, future research on social–ecological systems should include co-ordinated, long-term comparative projects across multiple sites to capture a full spectrum of variations (Liu et al. 2007). Thus, we also argue that wisely designed comparative studies of places can be used to test hypotheses about how different approaches to societal steering depend on context. The European continent’s variation in all dimensions of landscapes provides ample opportunity for multiple case studies of landscapes (Angelstam et al. 2013c). This approach provides benefits in terms of both producing context-dependent knowledge (e.g., Flyvbjerg 2011), comparative studies of different contexts (Elbakidze et al. 2013b), and meta-analyses (e.g., Angelstam et al. 2004b). Case studies and statistical methods are thus not conflicting but complementary (Flyvbjerg 2011).

The political and cultural diversity of the European continent (Berend 1986; Bugajski and Pollack 1989; Chirot 1989; Best 2009) presents a unique opportunity to develop a suite of local place-based learning processes. In Europe’s north, the Baltic Sea and Barents Sea Regions are two good examples of the need for knowledge production, learning and collaboration toward adaptive governance and integrated land-use planning of natural resources (Elbakidze et al. 2007). Examples of current issues linked to natural resources include how to intensify forestry in Russia (Holopainen et al. 2006), restore forest biodiversity in Sweden (Angelstam et al. 2011b), define conservation targets for aquatic ecosystems (Degerman et al. 2004), develop destinations for tourism (Saarinen 2003), make rural areas attractive for inhabitants (Briedenhann and Wickens 2004), and enhance urban green infrastructures for human health (Grahn and Stigsdotter 2010).

## Electronic supplementary material

Below is the link to the electronic supplementary material.

Supplementary material 1 (PDF 170 kb)
