# Supplementary material for: Measurement, Collaborative Learning and Research for Sustainable Use of Ecosystem Services: Landscape Concepts and Europe as Laboratory
Source: Ambio. 2013 Mar 10;42(2):129–45. doi: 10.1007/s13280-012-0368-0 (PMC3593029; doi:10.1007/s13280-012-0368-0)
Supplement: Supplementary file 1 — Supplementary material 1 (PDF 170 kb) [file 13280_2012_368_MOESM1_ESM.pdf]

Electronic Supplementary Material

Measurement, development and research for sustainable use of ecosystem services: landscape concepts and Europe as laboratory

Per Angelstam, Michael Grodzynskyi, Kjell Andersson, Robert Axelsson, Marine Elbakidze, Alexander Khoroshev, Ivan Kruhlov, Vladimir Naumov

Table S1 List of scholarly synonymous concepts developed to capture the need to consider both social and ecological systems in the context of knowledge production and learning to support implementation of policies about sustainable development and sustainability.

Term	Reference	Data collection network
Social-ecological system	Berkes and Folke (1998), Gunderson and Holling (2002), Berkes et al. (2003)	
Coupled human-environment systems	Turner et al. (2003); GLP (2005)	Global Lands Project
Coupled socio-environment system	Dearing et al. (2006)	
Coupled Natural and Human Systems (CNH)	http://www.nsf.gov/pubs/2010/nsf10612/nsf10612.pdf	
Coupled human-environment systems (CHANS)	Liu et al. (2007), McConnell et al. (2011)	
Socioecological system	Redman et al. (2004), Haberl et al. (2006)	ILTER; Long-Term SocioEcological Research (LTSER)

References

Berkes, F., J. Colding and C. Folke. 2003. *Navigating social–ecological systems: building resilience for complexity and change*. Cambridge: Cambridge University Press.

Berkes, F and C. Folke. 1998. *Linking Social and Ecological Systems*. Cambridge: Cambridge University Press.

GLP (Global Land Project). 2005. *Science plan and implementation Strategy*. IGBP Report No. 53/IHDP Report No. 19, 64pp. Stockholm: IGBP Secretariat.

Dearing, J.A., R.W. Battarbee, R. Dikau, I Larocque, and F. Oldfield. 2006. Human-environment interactions: learning from the past. *Regional Environmental Change* 6(1-2): 115-123.

Gunderson, L. and C.S. Holling. 2002. *Panarchy: understanding transformations in human and natural systems*. Washington, DC.: Island Press.

Haberl, H., V. Winiwarter, K. Andersson, R.U. Ayres, C. Boone, A. Castillo, G. Cunfer, M. Fischer-Kowalski et al. 2006. From LTER to LTSER: conceptualizing the socioeconomic dimension of long-term socioecological research. *Ecology and Society* 11(2): 13.

Liu, J., T. Dietz, S.R. Carpenter, M. Alberti, C. Folke, E. Moran, A.N. Pell, P. Deadman, T. et al. 2007. Complexity of coupled human and natural systems. *Science* 317: 1513-1516.

McConnell, W.J. J.D.A. Millington, N.J. Reo, M. Alberti, H. Asbjornsen, L.A. Baker, N. Brozović, L.E. Drinkwater et al. 2011. Research on Coupled Human and Natural Systems (CHANS): Approach, Challenges, and Strategies. *Bulletin of the Ecological Society of America* 92: 218–228.

Redman, C., M.J. Grove, and L. Kuby. 2004. Integrating Social Science into the Long Term Ecological Research (LTER) Network: Social Dimensions of Ecological Change and Ecological Dimensions of Social Change. *Ecosystems* 7(2): 161-17

Turner, R.K., J. Paavola, P. Cooper, S. Farber, V. Jessamy, and S. Georgiou. 2003. Valuing nature: lessons learned and future research directions. *Ecological Economics* 46(3): 493–510.

Table S2 Examples of variables proposed or used to measure different dimensions of landscape, which are linked to biophysical natural, anthropogenic and intangible perceived landscape concepts, which together make up the coupled social-ecological interpretation.

Sustainability criteria	Landscape interpretations		
	Biophysical natural	Anthropogenic	Intangible perceived
Ecological	Elements of biodiversity including composition (species), structure (habitats) and function (processes) (e.g., Angelstam and Dönz-Breuss 2004; Brumelis et al. 2011); Biological production capacity (Andersson et al. 2012a); Degree of naturalness (Brumelis et al. 2011); Landscape's nature potentials (Mannsfeld 1979)	Energy consumption, greenhouse gases and Assessment of landscape functions (Bastian 1999)	
Economic	Tree species and age class distribution (Elbakidze et al. 2013a)	Number of employed between 20-64 year, Salary level, Business climate, Commuting and Tax base (Andersson et al. 2012a)	Total economic value profiles (e.g., Richnau et al. 2013); quiet areas (Andersson et al. 2012b)
Cultural		Cultural landscape, cultural heritage, cultural access, participation and consumption of culture (Axelsson et al. 2013)	Social capital (Axelsson et al. 2013); Landscape preference indices (Kaplan and Kaplan 1989)
Social			Democratic local government, green infrastructures for human wellbeing, human development, gender equity (Axelsson et al. 2013)<br>Level of education, Exposed for violence, Expected average life span, Sick leave and Demographic dependency (Andersson et al. 2012 a, b)

References

Andersson, K., P. Angelstam, R. Axelsson, M. Elbakidze, and J. Törnblom. 2012a. Connecting municipal and regional level planning: analysis and visualization of sustainability indicators in Bergslagen, Sweden. *European Planning Studies* doi: 10.1080/09654313.2012.737991.

Andersson, K., P. Angelstam, M. Elbakidze, R. Axelsson, and E. Degerman. 2012b. Green infrastructures and intensive forestry: need and opportunity for spatial planning in a Swedish rural-urban gradient. *Scandinavian Journal of Forest Research* 28(2): 143-165.

Angelstam, P., and M. Dönz-Breuss. 2004. Measuring forest biodiversity at the stand scale – an evaluation of indicators in European forest history gradients. *Ecological Bulletins* 51: 305-332.

Axelsson, R., P. Angelstam, E. Degerman, S. Teitelbaum, K. Andersson, M. Elbakidze, and M. Drotz. 2013. Social and cultural sustainability: interpretation, indicators, variables for measurement and vizualisation to support planning. *AMBIO* 42(2): 215–228.

Bastian, O. 1999. The assessment of landscape functions – one precondition to define management goals. *Ekologia (Bratislava).* Suppl 17. Pp. 19-33.

Brumelis, G., B.G. Jonsson, J. Kouki, T. Kuuluvainen, and E. Shorohova. 2011. Forest naturalness in northern Europe: perspectives on processes, structures and species diversity. *Silva Fennica* 45(5): 807-821.

Elbakidze, M., K. Andersson, P. Angelstam, G.W. Armstrong, R. Axelsson, F. Doyon, M. Hermansson, J. Jacobsson, and Yu. Pautov. 2013a. Sustained yield forestry in Sweden and Russia: how does it correspond to sustainable forest management policy? *AMBIO* 42(2): 160–173.

Kaplan, R., and Kaplan, S. 1989. *The experience of nature: A psychological perspective.* Cambridge: Cambridge University Press.

Mannsfeld, K. 1979. Die Beurteilung von Naturraumpotentialen als Aufgabe der geographischen Landschaftsforschung.[The appreciation of the natural environment as a potential object of geographical landscape research] *Petermanns Geogr. Mitt.* 123. Pp. 2-6.

Richnau, G., P. Angelstam, S. Valasiuk, L. Zahvoyska, R. Axelsson, M. Elbakidze, J. Farley, I. Jönsson, et al. 2013. Multi-faceted total economic value profiles of forest owner categories in South Sweden: the River Helge å catchment as a case study. *AMBIO* 42(2): 188–200.
